# Oral absorption of PEG-coated versus uncoated gold nanospheres: does agglomeration matter?

**DOI:** 10.1186/s12989-015-0085-5

**Published:** 2015-03-25

**Authors:** Georgia K Hinkley, Paul Carpinone, John W Munson, Kevin W Powers, Stephen M Roberts

**Affiliations:** Center for Environmental and Human Toxicology, University of Florida, Box 110885, Gainesville, FL 32611 USA; Major Analytical and Particle Analysis Instrumentation Center, University of Florida, Box 116400, Gainesville, FL 32611 USA; Particle Engineering Research Center, University of Florida, Box 116135, Gainesville, FL 32611 USA

**Keywords:** Agglomeration, Gastrointestinal uptake, Gold nanoparticles, *In vivo* characterization

## Abstract

**Background:**

Particle size is thought to be a critical factor affecting the bioavailability of nanoparticles following oral exposure. Nearly all studies of nanoparticle bioavailability focus on characterization of the primary particle size of the material as supplied or as dosed, and not on agglomeration behavior within the gastrointestinal tract, which is presumably most relevant for absorption.

**Methods:**

In the study reported here, snapshots of agglomeration behavior of gold nanospheres were evaluated *in vivo* throughout the gastrointestinal tract using transmission electron microscopy. Agglomeration state within the gastrointestinal tract was then used to help explain differences in gastrointestinal particle absorption, as indicated by tissue levels of gold detected using inductively coupled plasma mass spectrometry. Mice were dosed (10 mg/kg) with either 23 nm PEG-coated or uncoated gold nanospheres.

**Results:**

Transmission electron microscopy demonstrates that PEG-coated gold nanoparticles can be observed as primary, un-agglomerated particles throughout the gastrointestinal tract and feces of dosed animals. In contrast, uncoated gold nanoparticles were observed to form agglomerates of several hundred nanometers in all tissues and feces. Inductively coupled plasma mass spectrometry shows significantly higher levels of gold in tissues from animals dosed with PEG-coated versus uncoated 23 nm gold nanoparticles. Retention of particles after a single oral gavage was also very high, with all tissues of animals dosed with PEG-coated particles having detectable levels of gold at 30 days following exposure.

**Conclusions:**

Qualitative observation of these particles *in vivo* shows that dispersed PEG-coated particles are able to reach the absorptive tissues of the intestine while agglomerated uncoated particles are sequestered in the lumen of these tissues. However, the large differences observed for *in vivo* agglomeration behavior were not reflected in oral absorption, as indicated by gold tissue levels. Additional factors, such as surface chemistry, may have played a more important role than *in vivo* particle size and should be investigated further.

## Background

The projected market value for nanotechnology products is expected to exceed $3 trillion this year, with over 1,000 consumer goods currently available containing nanomaterials [1,2]. Applications for these materials that may lead to oral exposure are continuing to expand and currently include food additives, food packaging, pharmaceuticals, natural supplements and personal care products [3-7]. Many of these food additives and other products have been used for decades with micron-sized particles, however newer products are engineering smaller particles to increase product effectiveness. It is estimated that humans are orally exposed to micron and nano-sized particles at a rate of approximately 40 mg per day, mostly consisting of sub-micron sized silica and TiO_2_ [1,8]. Due to the increased use of nanomaterials in consumer products, it is important to assess the potential for toxicological interactions. As a first step, the factors influencing their gastrointestinal uptake must be understood.

Trends affecting gastrointestinal absorption of nanomaterials have been investigated largely with latex and polystyrene particles. These materials can easily be made to have fluorescent properties, making the localization of particles *in vivo* relatively easy. Studies with polystyrene have shown that primary particle size is an important determinant for location of gastrointestinal uptake [9,10]. Evidence has been provided that 50 nm and 100 nm particles can be taken up via enterocytes in the upper small intestine, while larger particles (up to 500 nm) are taken up via Peyer’s Patches (PPs) in the lower small intestine [1,10]. However, a study by Jani *et al.* provides evidence that larger particles, up to 1 μm were able to achieve PP uptake. Particles with a diameter of 1 μm or less were reportedly found to translocate to the mesenteric lymph nodes after PP uptake and eventually reached the spleen via lymphatic circulation. Particles smaller than 1 μm were then able to exit the spleen via unknown mechanisms, leaving the 1 μm particles sequestered there. Qualitative investigations of particle uptake in the gastrointestinal tract (GIT) have also been performed with gold nanoparticles (AuNPs), showing that passive uptake during cell turnover is a primary mechanism of absorption [11]. While these studies offer evidence for size-dependent mechanisms of uptake, they provide little quantitative data on the uptake of ingested particles.

Some studies have quantitatively investigated the oral absorption of nanomaterials (i.e., their bioavailability), but insight regarding the influence of size is confounded by incomplete particle characterization. No studies to date have analyzed *in vivo* particle characteristics *in situ* following oral exposure and related them to the extent of particle uptake. Changes in effective particle size are likely to occur *in vivo* within the gastrointestinal tract due to agglomeration, especially in the harsh pH of the stomach and strong ionic conditions throughout the gastrointestinal tract. As the effective particle size at the absorptive surfaces of the GIT increases with agglomeration, size-dependent uptake is likely to be influenced. Many studies associate their bioavailability results with characterization of the “as-dosed” material, but the *in vivo* agglomeration state of the nanoparticles has largely been ignored as a factor affecting particle uptake.

We report here the results of a study using transmission electron microscopy to relate snapshots of agglomeration behavior of gold nanoparticles in the GIT and to particle uptake. Gold was chosen for this study because it is electron dense and can be readily visualized by transmission electron microscopy (TEM), facilitating detection of particles in complex biological environments such as the gastrointestinal tract. It is also resistant to dissolution in physiological fluids (including the low pH of gastric fluids) and has a low background concentration in tissues. This allows the intact gold nanoparticle content of tissues to be measured in a relatively straightforward manner by determining gold concentrations by inductively coupled plasma mass spectrometry (ICP-MS).

It was hypothesized that polyethylene glycol-coated (PEG-coated) and uncoated 23 nm gold spheres would display contrasting agglomeration behavior following oral administration, with uncoated gold extensively agglomerating while the PEG-coated nanoparticles remained dispersed. We further hypothesized that agglomeration of uncoated gold nanoparticles would result in diminished uptake from the GIT as compared with PEG-gold. To test these hypotheses, a single gavage dose of either uncoated or PEG-coated gold nanoparticles to mice and the *in vivo* agglomeration state of each was observed, beginning in the stomach and continuing in each organ (small intestine, cecum, large intestine) until excretion. Uptake was evaluated by measuring gold concentrations in tissues over time following gavage. This study represents, to the best of our knowledge, the first attempt to relate particle uptake behavior *in vivo* to agglomeration state as it exists at potential absorptive sites within the GIT.

## Results

### Particle characterization

**Figure 1:**
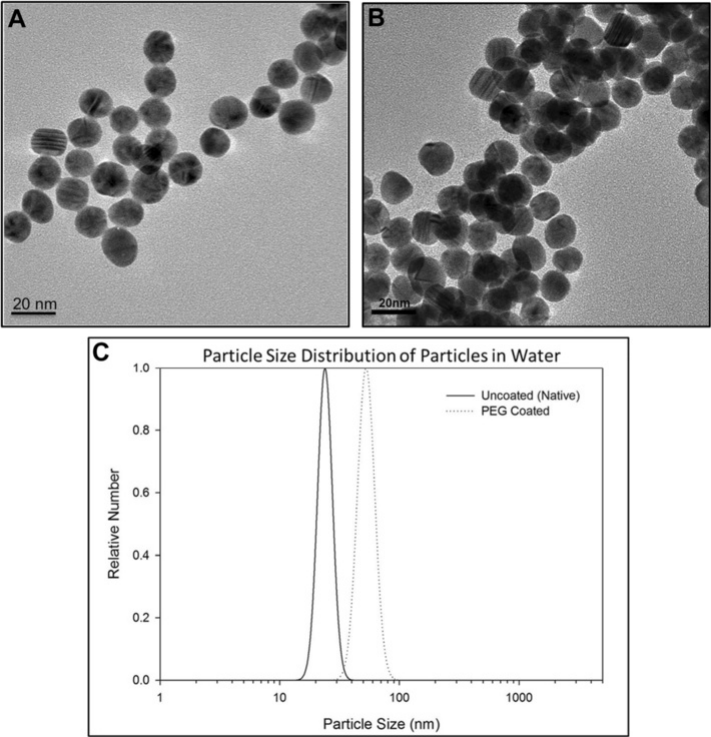
**Particle characterization: TEM and dynamic light scattering**. Transmission electron micrograph of PEG-coated **(A)** and uncoated **(B)** gold nanospheres. One-hundred particles were measured for each particle type to determine the average primary particle size to be 14 ± 2 nm. Dynamic light scattering particle size distributions of the as dosed PEG-coated and uncoated particles **(C)** measured the hydrodynamic diameter of the particles to be 45 ± 2 nm and 23 ± 6 nm, respectively.

The TEM analysis indicated that the synthesized gold nanoparticles were spherical with an average primary particle size of 14 nm ± 2 nm (Figure 1A, B). The particle surface area was calculated to be 22.2 m^2^/g. To estimate the surface area per mass, the surface area per particle was divided by the mass per particle. The surface area per particle was calculated with the assumption that the particles are spherical, using the equation for the surface area of a sphere (SA = 4**π**r^2^). The mass per particle was calculated with the assumption that the particles are spherical using the density of gold (19.3 g/cm^3^) and the equation for the volume of a sphere (V = 4/3* **π**r^3^)**.** Based on the mass concentration of the dosing solution (1.5 mg/mL as measured using ICP-MS following aqua regia digestion), and the mass per particle calculations, the dosing solution contained 5.4 x 10^13^ particles/ml. Dynamic light scattering (DLS) was used to determine hydrodynamic diameter of the as dosed materials, revealing the uncoated particlesto have a distribution centered around 23 nm ± 6 nm while the PEG-coated particles had a distribution centered around 45 nm ± 2 nm (Figure 1C).

### Particle agglomeration and dissolution in a simulated gastric environment

**Figure 2:**
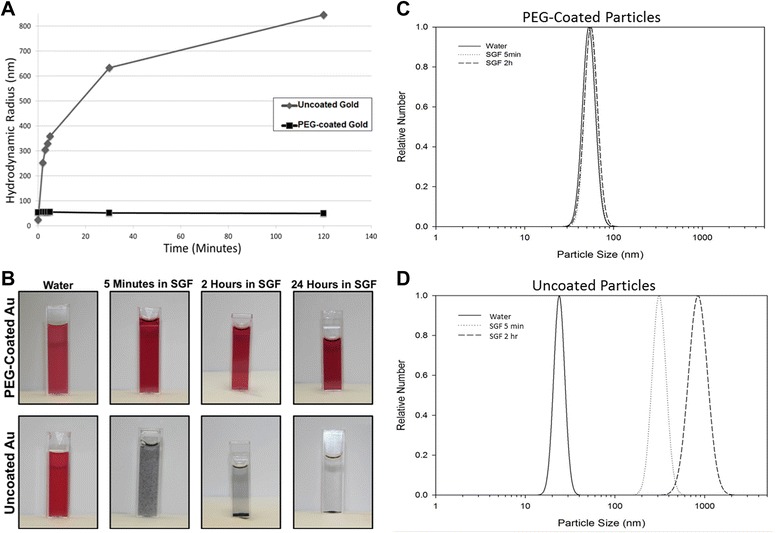
**PEG-coated and uncoated 23 nm gold nanoparticle behavior in simulated gastric fluid.** Dynamic light scattering was used to show the agglomeration behavior of PEG-coated and uncoated 23 nm gold nanoparticles in simulated gastric fluid during 2 hours of incubation at 37**°**C**.** Figure 2 **A** shows both particle types over the 2 hour time course; uncoated gold nanoparticles agglomerate immediately while PEG-coated particles remain well dispersed in simulated gastric fluid. Figure 2 **B** shows photographs of PEG-coated and uncoated gold particles in water and in simulated gastric fluid after 5 minutes, 2 hour and 24 hours. As seen using DLS, PEG-coated particles remain well dispersed and appear as they do in water while uncoated particles agglomerate extensively. The particle size distributions for the particles in water and in SGF at 5 minutes and 2 hours are also shown for PEG-coated (Figure 2 **C**) and uncoated particles (Figure 2 **D**).

Both PEG-coated and uncoated particle suspensions were stable in water. However, when placed in simulated gastric fluid (SGF), the uncoated gold particles agglomerated immediately, reaching sizes over 800 nm in two hours, while the hydrodynamic diameter of the PEG-coated particles remained unchanged even after 24 hours in SGF (Figure 2A). The difference in agglomeration behavior can also be observed in photographs of the two particles types in water and in simulated gastric fluid after 5 minutes, 2 hours and 24 hours (Figure 2B). Little change is observed for PEG-coated particles even after 24 hours in SGF, while the uncoated particles visibly change after 5 minutes in SGF and have agglomerated so extensively after 2 hours that they have settled out of suspension. Particle size distributions of the particles in water and in simulated gastric fluid at 5 minutes and 2 hours are shown in Figure 2C (PEG-coated particles) and Figure 2D (uncoated particles). To confirm the absence of dissolution of gold particles under gastric conditions, uncoated particles were incubated in SGF for 5 hours. Measurement of gold in ultrafiltrate following incubation found that only 0.003% of the mass of gold nanoparticles incubated was present as gold ions (data not shown).

### Oral bioavailability and particle uptake into tissues

**Figure 3:**
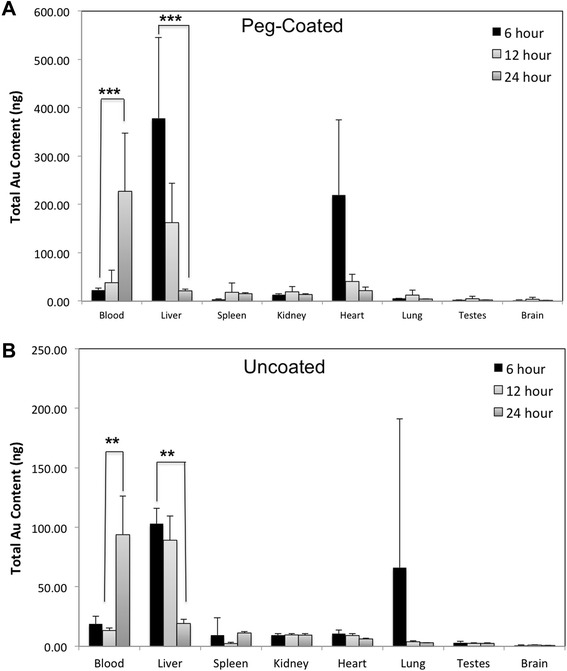
**Tissue concentrations of PEG-coated** ^**1**^ **(A) and uncoated (B) 23 nm gold spheres 6, 12 and 24 hours post-gavage.** Total gold measured in tissues and blood six, twelve and twenty-four hours following oral gavage of PEG-coated **(A)** and uncoated **(B)** 23 nm AuNPs suspended in water. Gold was measured using ICP-MS. Data are presented as average ± SD, n = 5. Statistical significance: * indicates a p-value ≤ 0.05, ** indicates a p-value ≤ 0.01 and *** indicates a p-value ≤ 0.005. ^1^Particle size given for PEG-coated particles is reflective of the gold core, the hydrodynamic diameter of the PEG-coated particles was determined to be 45 nm.

Mice were orally gavaged with either PEG-coated or uncoated 23 nm gold spheres (10 mg/kg in water), and tissues and feces were collected and analyzed using ICP-MS to quantify oral uptake of PEG-coated and uncoated 23 nm gold spheres. Significantly higher levels of gold were detected in the liver and heart of animals dosed with PEG-coated versus uncoated particles at six hours post-gavage (Table 1). Twenty-four hours post-gavage, higher gold concentrations were observed for several tissues of animals dosed with PEG-coated vs. uncoated gold nanoparticles: blood, spleen, kidney, heart, lung and brain (Table 1). Differences in the amount of gold detected in liver at six hours were no longer significant at 24 hours post-gavage, suggesting redistribution of absorbed particles during the first 24 hours following dosing. Redistribution patterns of gold for animals dosed with PEG-coated and uncoated particles are shown in Figure 3A and B, respectively, at 6, 12 and 24 hours post-gavage. The level of gold in blood increased over the first 24 hours, while gold levels in the liver significantly decreased over this time period for mice dosed with either particle type.

**Table 1:** **Total gold content of tissues 6, 12 and 24 hours following oral exposure to gold nanoparticles**

	**6 Hour**		**12 Hour**		**24 Hour**	
	**Total Au content (ng)**		**Total Au content (ng)**		**Total Au content (ng)**	
	**(SD)**		**(SD)**		**(SD)**	
	**PEG-coated**	**Uncoated**	**PEG-coated**	**Uncoated**	**PEG-coated**	**Uncoated**
**Blood**	22.2	18.7	38.1	13.2	227.1*	93.8
	(5.0)	(6.7)	(26.2)	(2.2)	(120.6)	(32.5)
**Liver**	378.1**	103.0	162.3	89.1	21.2	19.1
	(166.9)	(12.1)	(81.8)	(20.6)	(3.7)	(3.7)
**Spleen**	3.8	9.4	18.2	2.2	15.3**	11.1
	(1.0)	(14.6)	(18.9)	(0.8)	(2.3)	(1.4)
**Kidney**	13.1	9.3	19.3	9.5	13.6***	9.3
	(2.5)	(1.3)	(10.8)	(1.1)	(1.9)	(1.2)
**Heart**	219.1**	10.4	40.6**	9.0	21.6***	6.1
	(155.5)	(3.3)	(15.0)	(1.7)	(6.9)	(0.7)
**Lung**	5.5	66.1	12.4	3.7	4.4***	2.9
	(0.4)	(124.9)	(10.0)	(1.0)	(0.8)	(0.1)
**Testes**	2.5	3.1	4.9	2.4	2.3	2.4
	(0.2)	(1.1)	(4.8)	(0.3)	(0.5)	(0.4)
**Brain**	1.8	0.9	3.8	1.0	1.5**	0.6
	(0.3)	(0.1)	(3.6)	(0.3)	(0.5)	(0.1)

Asterisks indicate statistically significant differences in total gold content for tissues from mice dosed with PEG-coated versus uncoated gold particles for each time point. * indicates a p-value ≤ 0.05, ** indicates a p-value ≤ 0.01 and *** indicates a p-value ≤ 0.005. For all groups, averages are based on n = 5, and standard deviations are shown in parentheses below each average.

**Figure 4:**
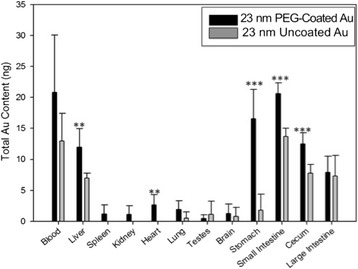
**Tissue concentrations of PEG-coated** ^**1**^ **and uncoated 23 nm gold spheres 30 days post-gavage.** Total gold measured in tissues and blood thirty days following oral gavage of PEG-coated and uncoated 23 nm AuNPs suspended in water. Gold was measured using ICP-MS. Data are presented as average ± SD, n = 5. Statistical significance: * indicates a p-value ≤ 0.05, ** indicates a p-value ≤ 0.01 and *** indicates a p-value ≤ 0.005. ^1^Particle size given for PEG-coated particles is reflective of the gold core, the hydrodynamic diameter of the PEG-coated particles was determined to be 45 nm.

The retention of absorbed particles 30 days following a single oral gavage was also investigated. Gold was detected in all tissues of mice treated with PEG-coated gold particles and many tissues of mice treated with uncoated particles. These data demonstrate high retention of absorbed particles, even after an acute exposure (Figure 4). It is very interesting to note that there remained a relatively high amount of gold in all tissues of the GIT (stomach, small intestine, cecum and large intestine) at 30 days post-gavage. For Table 1 and Figures 3 and 4, it is important to emphasize that the data are expressed in terms of total gold per tissue in nanograms. While significant differences were observed between the particle types, the values detected in the tissues of animals given either dosing solution are more than three orders of magnitude less than the total administered dose (300 μg): demonstrating very poor oral absorption of either particle type.

### *In vivo* agglomeration

**Figure 5:**
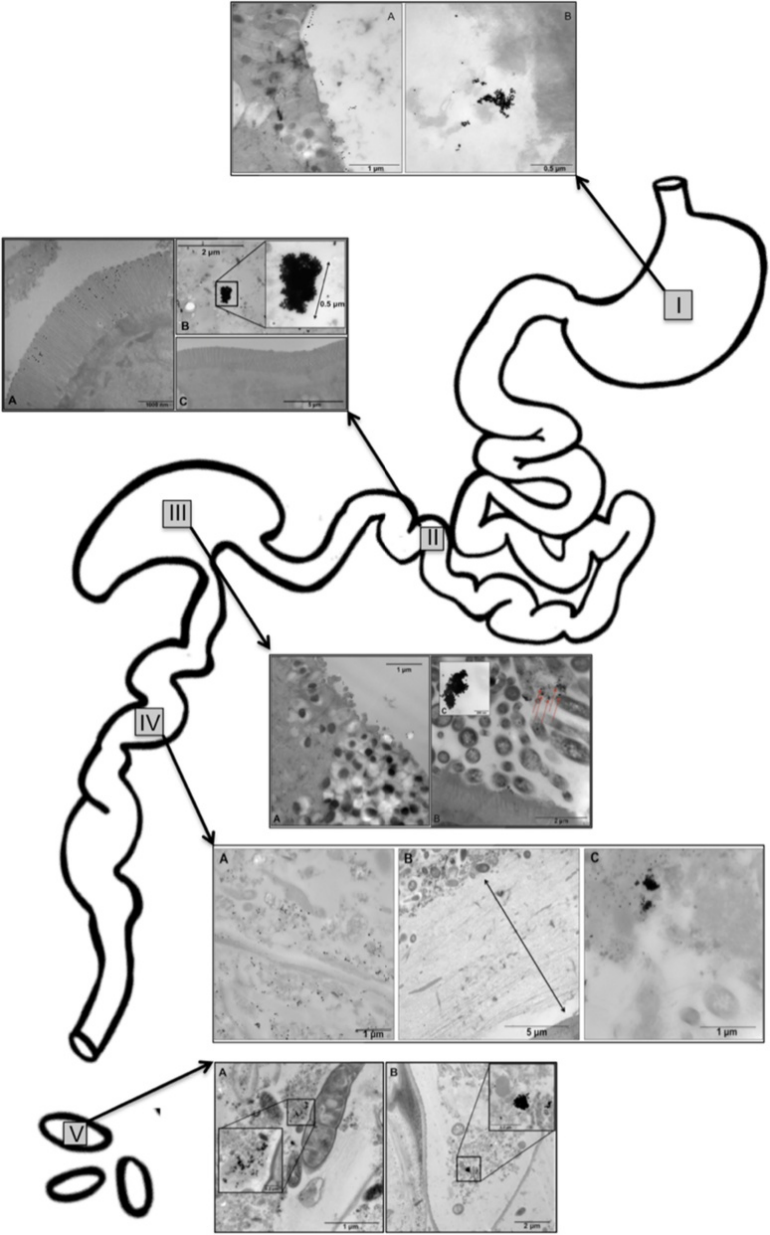
**TEM images of PEG-coated and uncoated 23 nm gold spheres throughout the gastrointestinal tract.** TEM images of gold nanospheres found throughout the gastrointestinal tract of a mouse following oral gavage: in the stomach **(I)** 10 minutes post-gavage (PG), small intestine **(II)** 2.5 hours PG, cecum **(III)** 4 hours PG, large intestine **(IV)** 5 hours PG and feces (V) 24 hours PG. Micrographs from animals gavaged with PEG-coated particles are shown in panel A for all five insets, uncoated particles are shown in panel B for all insets except IV (large intestine) in which uncoated particles are shown in panel C. As seen in snap shots throughout the GIT, PEG-coated particles remain well dispersed while uncoated particles appear to agglomerate extensively, beginning immediately after dosing, as seen in the stomach **(I)**. In addition to a contrast in agglomeration behavior, PEG-coated particles are frequently seen adjacent to the absorptive surfaces within the GIT **(I-III)** while uncoated particles are only observed in the tissue lumen, distal from the surface **(I-IV)**. As seen in the small intestine **(II)**, panel C shows the microvilli of a mouse dosed with uncoated particles being entirely void of particles. In image IV, large intestine, the thick mucin layer can be seen in panel B, potentially explaining why both PEG-coated and uncoated particles were only observed in the lumen of the tissue.

**Figure 6:**
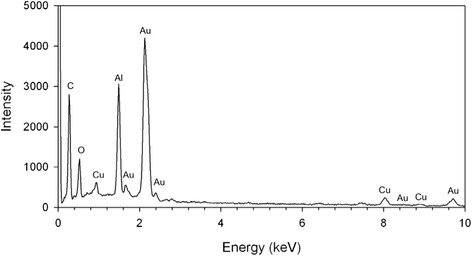
**Energy-dispersive X-ray spectroscopy.** Energy-dispersive X-ray spectroscopy of 23 nm uncoated gold nanoparticles in the cecum 4 hours following oral gavage. The presence of peaks from Al and Cu originate from the specimen holder and grid respectively.

Mice were dosed as previosuly described and the *in vivo* agglomeration behavior of the particles was investigated throughout the GIT (stomach, ileum/PP region, cecum, large intestine and feces) using TEM. For these experiments, tissue and lumen contents were fixed to allow visualization of the location of particles in the lumen relative to cells lining the GIT, as well as to prevent agglomeration artifacts during sample preparation. The impact of TEM processing on particle suspension stability within tissues cannot be predicted, however, it is likely much less dramatic than the artifacts that are known to exist for TEM preparation of particle suspensions in water (i.e. drying effect). Figure 5 shows a diagram of the GIT with five inset images (I-V) representing snapshots of agglomeration behavior of PEG-coated and uncoated particles in the stomach (I), small intestine (II), cecum (III), large intestine (IV) and feces (V). PEG-coated gold particles are frequently observed as primary/un-agglomerated particles near the tissue surface of the stomach (Figure 5-IA). In contrast, the majority of the observed mass dose of uncoated gold particles was found only in the lumen of the stomach as agglomerates (Figure 5-IB). This pattern was also seen in the Peyer’s patch region of the ileum; unagglomerated PEG-coated particles in Figure 5-IIA can be seen interacting with microvilli adjacent to the dome region of a PP, while uncoated gold particles were primarily located in the lumen of the ileum as large agglomerates, approximately 4 μm from the tissue surface (Figure 5-IIB). Figure 5-IIC shows the ileal microvilli of a mouse dosed with uncoated gold particles at the same time point, demonstrating that the tissue surface was observed to be void of particles. The agglomeration behavior in the cecum was similar; PEG-coated particles were found near the tissue surface as primary particles (Figure 5-IIIA) and uncoated particles were observed as agglomerates in the cecum lumen, 5 μm from the tissue surface (Figure 5-IIIB). The pattern changed somewhat in the colon, where both particle types were found only in the lumen of the large intestinal approximately 10 μm from the tissue surface (Figures 5-IVA and C). However, as seen in the other GIT organs, the agglomeration behavior in the large intestine remains as previously described. Figure 5-IVB shows the thick mucin layer present in the large intestine which may have prevented either particle type from migrating to the tissue surface. Finally, agglomeration behavior was investigated in excreted feces. The feces from mice dosed with PEG-coated particles contained loose agglomerates, likely the result of dehydration in the colon, as well as several primary particles (Figure 5-VA), while the majority of particles in the feces of mice dosed with uncoated gold were found as densely packed agglomerates (Figure 5-VB). Composition of the imaged particles was confirmed to be gold using energy-dispersive X-ray spectroscopy (Figure 6).

## Discussion

While the data presented here show significant differences for gold content in tissues, suggesting higher absorption of PEG-coated versus uncoated gold particles, both particle types had very low absorption with less than 1% of the administered dose being found in tissues, cumulatively. Other groups have demonstrated low gastrointestinal absorption of gold nanoparticles as well. Schleh and coworkers [12] administered a single oral gavage dose of TPPMS-coated ^198^Au nanoparticles to rats, with nanoparticle sizes ranging from 1.4 to 200 nm; the percent dose recovered in feces and found in GIT contents was >99.6 for all particle sizes, suggesting that oral bioavailability was very low. Although nanoparticles eliminated in the feces could theoretically include particles that were absorbed and eliminated in the bile, other studies of gold nanoparticles administered by intravenous injection have shown that biliary excretion of gold nanoparticles is minimal, around 5% for either PEG-coated or uncoated small (<10 nm) gold nanoparticles in rats [13,14]. The absence of significant biliary excretion indicates that gold nanoparticles appearing in feces are reasonable estimates of unabsorbed material.

The very low oral bioavailability of gold nanoparticles observed in rats by Schleh and coworkers over a wide nanoparticle size range is consistent with results shown here. Extensive agglomeration of uncoated gold throughout the gastrointestinal tract observed in this study could explain the low absorption of the uncoated gold nanoparticles presented in this study, as well as the low bioavailability of the particles studied by Schleh and others. However, the poor absorption of the PEG-coated particles was more surprising. Simulated gastric incubations indicated rapid agglomeration of uncoated gold nanoparticles, while PEG-coated gold nanoparticles remained dispersed. This difference in agglomeration was also observed through snapshots of *in vivo* agglomeration behavior using TEM. PEG-coated 23 nm gold nanoparticles were observed as un-agglomerated primary particles throughout the GIT, and were frequently found adjacent to the absorptive surfaces of the GIT tissues. In contrast, uncoated 23 nm gold nanoparticles were found almost entirely as large agglomerates located away from GIT epithelial surfaces. TEM sections from the GIT, while observing many more particles in the lumen near epithelial lining in animals dosed with PEG-coated particles, showed almost no particles absorbed into the GIT tissues in animals given either uncoated or PEG-coated particles.

The presence of a thick protective layer of mucin coating the GIT is likely responsible for limiting access of the agglomerated uncoated particles to these tissue surfaces. Particle size has been shown to be influential in determining the ability of a particle to move through the mucin layer, which can be as thick as 100 μm in humans and 30–50 μm in rats [15,16]. Goblet cells maintain this protective mucus with complete turnover of the mucin layer every 4–6 hours [17,18]. For particles to reach absorptive surfaces they must transit the entire mucin layer during this time frame. Smaller particles move through the layer most rapidly, and based on experimental data, particles ≥ 1 μm are immobile in mucin [16]. Other groups have shown that PEG surface coating is favorable for efficient mucin movement, probably due to neutral zeta potential [19]. It is important to note however, that similar to the importance of *in vivo* size measurements, it would be difficult to draw conclusions about the impact of surface charge without *in vivo* characterization*.* The PEG surface coating used in this study may have aided the increased uptake of those particles; however, it is also likely that the large agglomerate sizes of the uncoated particles played a role in inhibiting their uptake.

Another important consideration for the role of the protective mucin layer is that the PP region of the small intestine is devoid of goblet cells and has very diminished mucus production, allowing interaction of the GIT immune cells with ingested materials [20]. Consistent with this, a previous study with fluorescent polystyrene particles has shown PP uptake of 1 μm particles [10]. In the study reported here, we did not see large agglomerates of uncoated gold nanoparticles interacting with the PP regions of the small intestine. However, the TEM analysis used in this study served to provide a snapshot of agglomeration behavior throughout the GIT; without quantitative analysis, this type of interaction with gold nanoparticle agglomerates cannot be ruled out.

While PEG-coating may have potentially been advantageous to transit the thick mucin layer, there is evidence that PEG-coating can decrease macrophage uptake of iron particles and that longer chain lengths increase uptake hindrance [21]. Other groups have shown that coating nanoparticles with various materials (food or immune-related) can influence their uptake into cells and the impact of PEG-coating on intestinal absorption should be investigated further [22-24]. In addition, PEG-coating undoubtedly changed the surface charge (not measured here) of the particles compared to an uncoated gold surface. This change in surface charge may have further enhanced the difference in protein corona between the particle types. While PEG-coating is a mechanism to prevent agglomeration, certain aspects of the particle surface chemistry will be changed, possibly preventing the adsorption of surface macromolecules that facilitate absorption.

## Conclusions

When evaluating particle characteristics that influence their uptake, agglomeration state within the GIT must be assessed and taken into consideration. By focusing on particle behavior at the site of absorption more meaningful conclusions can be reached about factors influencing oral bioavailability as compared to studies that only consider the “as dosed” characterization data. Gold nanoparticles can be readily detected and their agglomeration behavior and surface properties can be manipulated, making them valuable tools with which to explore factors controlling uptake from the gut. This study suggests that agglomeration behavior and effective particle size within the gastrointestinal tract may not be a significant factor for the oral bioavailability of gold nanomaterials, however, for other materials it may play a larger role and should not be discounted as a factor affecting bioavailability of nanomaterials. Additional studies, quantitatively analyzing events occurring *in situ* at absorptive surfaces of the gut, will be needed to better understand the relationship of particle properties and nanoparticle bioavailability.

## Methods

### Materials

All reagents used for particle synthesis of PEG-coated and uncoated gold nanospheres were purchased from Fisher Scientific (Pittsburgh, PA, USA). All materials used for transmission electron microscopy tissue processing were purchased from Electron Microscopy Sciences (Hatfield, PA, USA). All components of the simulated gastric fluid, nitric acid, hydrochloric acid, and hydrogen peroxide were purchased from Fisher Scientific (Pittsburgh, PA, USA). Hydrophilic PTFE (polytetrafluoroethylene) 0.22 μm filters for ICP-MS analysis were purchased from MilliPore™ (Billerica, MA, USA).

### Nanoparticle preparation and characterization

Spherical gold particles with a nominal particle size of 23 nm were synthesized by citrate reduction of gold chloride as described previously [25]. These particles were washed and concentrated by centrifugation. A portion of the uncoated particles were then reacted with excess thiol-terminated polyethylene glycol (5 kDa) to achieve PEG-coating. All particles were characterized with a Tecnai™ Spirit transmission electron microscope (FEI Company, Hillsboro, OR, USA), operated at 110 keV, to verify particle size and shape. Dynamic light scattering was used to determine hydrodynamic diameter and particle size distribution on a Brookhaven ZetaPlus instrument (Brookhaven Instruments Corp., Holtsville NY). Values indicated for particle size distributions are average diameter ± reproducibility of the measurement.

### Simulated gastric fluid incubations and dissolution

Characterization of 23 nm uncoated gold particles in simulated gastric fluid was performed using dynamic light scattering on a Brookhaven ZetaPlus instrument (Brookhaven Instruments Corp., Holtsville NY). Simulated gastric fluid (SGF) was prepared as previously described [26]. Particle dissolution was investigated after 5 hours in simulated gastric fluid at 37°C. Particles were removed by high speed centrifugation and the supernatant was tested for gold ions using inductively coupled plasma mass spectrometry on a Thermo Electron X Series II (Thermo Scientific, West Palm Beach, FL, USA).

### Animal housing and nanoparticle administration

Adult male (25–30 g) ICR outbred mice were purchased from Harlan Laboratories (Pratville, AL). Prior to dosing, mice were housed in cages (five mice per cage), with controlled light/dark cycles of 12 hours (08:00–20:00 light). Temperature (18–26°C) and humidity (30–70%) were also controlled. Animals were fed a standard diet (Tekland Rodent Diet 7912, Harlan Laboratories) and were fasted 10 hours prior to oral gavage and 2 hours post oral gavage; animals always had free access to water. Mice were given an oral gavage of 10 mg/kg of either PEG-coated or uncoated 23 nm gold nanoparticles in 200 μl of MilliQ™ water. The dose of 10 mg/kg was selected as a mid-range dose based on the literature, with other studies ranging from less than 1 mg/kg to 300 mg/kg. Actual administered dose was not quantified for each animal. Dose was estimated using a simulated gavage injection to estimate retention of dosing solution; the administered dose ranged from 99.5 to 101% of the desired dose. Following oral gavage, mice were placed in glass metabolism cages for collection of samples at the 6, 12 and 24 hour time points, while mice dosed for the 30 day time point were kept in metabolism cages for 5 days and then returned to standard housing for the duration of the experiment. Urine and feces were collected from metabolism cages. For ICP-MS analysis, mice were euthanized at 6, 12, 24 hours and 30 days post oral gavage using carbon dioxide inhalation and cervical dislocation. Tissues and blood (cardiac puncture) were collected following euthanasia and acid digested for ICP-MS analysis. For TEM analysis, mice were euthanized by carbon dioxide inhalation and cervical dislocation and tissues were collected at various time points (details below). This study was approved by the Institutional Animal Care and Use Committee, and all animals were treated humanely according to criteria provided in the NIH “Guide for the Care and Use of Laboratory Animals”.

### Transmission electron microscopy

For the TEM analysis portion of this study, a total of 10 animals were used. One animal was investigated for each pairing of sample type (stomach, small intestine, cecum, large intestine and feces) and particle solution (uncoated and PEG-coated gold spheres). For the small intestine investigation, the PP region was selected due to evidence that this region may be more important for uptake of “larger particles” [1,10]. Specific tissue processing details for each sample type are described below.

#### Stomach and cecum tissue processing

Fasted animals were dosed (10 mg/kg) with either a 23 nm PEG-coated or uncoated gold particle solution. Five minutes and 4 hours following oral administration, for stomach and cecum respectively, animals were euthanized with carbon dioxide inhalation. Following euthanasia, a large ventral incision was made to expose the abdominal cavity and heart. The hepatic portal vein was cut to be utilized as a drain and then a 24 gauge catheter was inserted into the left ventricle of the heart and clamped in place. The mouse was then perfused with 0.1 M sodium cacodylate buffer (0.1 M cacodylate buffer, 2 mM MgCl_2_,1 mM CaCl_2_, 2.5% NaCl, pH 7.24), continuing for five minutes after the hepatic drain appeared to be blood-free. The perfusion was then switched to a 2% glutaraldehyde/ 4% paraformaldehyde solution in 0.1 M sodium cacodylate buffer. Following complete perfusion, the stomach and cecum were dissected and placed into 15 mL of fresh 2% glutaraldehyde/ 4% paraformaldehyde fixative in 0.1 M sodium cacodylate buffer. Additional fixative was then injected slowly into the lumen of the stomach via the duodenum and lower esophageal sphincter using a syringe. Samples remained at room temperature (25°C) for 1 hour and were then held at 4°C overnight. The following morning, samples were transferred into fresh 0.1 M sodium cacodylate buffer and microwaved at 220 watts (PELCO Biowave Microwave Processor, Ted Pella Inc, Redding, CA, USA), under vacuum (15–20 mmHg), for 30 seconds, followed by 1 minute at room temperature; this was repeated twice with fresh buffer. Next, the stomach was gently cut with a scalpel and slices were placed on glass slides and embedded in warm agarose while sitting on ice. After the agarose was set and became gelatinous, samples were returned to 0.1 M sodium cacodylate buffer. Buffer was then replaced with a 2% OsO_4_ solution in 0.1 M sodium cacodylate buffer and then microwaved (180 Watts, lower wattage used for OsO_4_ only) under vacuum for 30 seconds, followed by 3 minutes at room temperature. Osmium tetroxide solution was removed and samples were placed in double distilled water (ddH_2_O) and microwaved with vacuum for 30 seconds, followed by 1 minute at room temperature; this was repeated twice with fresh ddH_2_O. Samples were then introduced to an ethanol series (in water) for dehydration: 25%, 30%, 40%, 50%, 60%, 75%, 80%, 90% and 100% twice. Samples were microwaved for 45 seconds (without vacuum), followed by 5 minutes at room temperature for each step. Ethanol was then replaced with 100% acetone and microwaved (without vacuum) for 45 seconds, followed by 5 minutes at room temperature; this was then repeated once with fresh 100% acetone. Next, samples were moved to a Spurr’s resin series (in acetone): 25%, 30%, 40%, 50%, 60%, 70%, 80%, 90% and 100% twice. Spurr’s resin was supplemented with 100 μl/10 ml of Z-6040, used as a primer, and 2-dimethylaminoethanol (DMAE), used as an accelerator. During each step of the Spurr’s series, samples were microwaved for 3 minutes, followed by 20 minutes at room temperature; samples in 100% Spurr’s were left at room temperature for 1 hour. Samples in 25%–60% Spurr’s were microwaved without vacuum, while samples in 70%–100% Spurr’s were microwaved under vacuum. Samples were left in 100% Spurr’s, covered with parafilm and placed on a rocker overnight at room temperature. The next morning, samples were moved to a resin mold and placed in fresh 100% supplemented Spurr’s resin. Molds were vacuumed to remove air bubbles and then placed at 60°C for 72 hours to ensure complete resin hardening.

#### Ileum (PPs), large intestine and feces processing

Due to the small size of these tissues, samples were collected immediately following euthanasia and did not require whole animal perfusion. Animals were dosed (10 mg/kg) with either 23 nm PEG-coated or uncoated gold spheres and then tissues were collected. Ileum samples were collected 2.5 hour post oral gavage, colon samples were collected at 5 hour post oral gavage and feces were collected 24 hours post oral gavage. Samples (~2 mm in length) were placed directly into a 2% glutaraldehyde/ 4% paraformaldehyde solution in 0.1 M sodium cacodylate buffer and processed as described above for stomach and cecum. The only difference being that these samples did not require agarose embedding, due to their smaller size.

#### Sectioning, staining, imaging

Samples were sectioned at 70–100 nm using a LEICA Ultracut UCT microtome (Leica Microsystems, Wetzlar, Germany). Sections were collected on uncoated 3 mm copper grids and stained with uranyl acetate and lead citrate before viewing on a Tecnai™ Spirit transmission electron microscope (FEI Company, Hillsboro, OR, USA) operated at 110 keV.

### Energy-dispersive X-ray Spectroscopy

Analysis of the elemental composition was performed on a section of cecum from a mouse treated with uncoated gold by use of a JOEL6335F SEM (JOEL USA, Inc, USA) operated at 15 kV. This instrument was equipped with an Oxford ISIS EDS system (Oxford Instruments plc, UK).

### Inductively coupled plasma mass spectrometry

The Thermo Electron X Series II (Thermo Scientific, West Palm Beach, FL, USA) was used for all ICP-MS analyses, with indium as an internal standard. The LOD was 0.1 ppb, with an LOQ of 1 ppb. Procedural blanks were run in conjunction with all samples and subtracted as background. All groups (two particle dosing groups and four time points) were run with an n = 5. One vehicle-control group (water only, 24 hours), n = 5, was used to measure background gold. The gold detected in all control animals was below the level of quantitation (<1 ppb in any single tissue). The total number of animals used for bioavailability measurements was 45 (n = 5 for controls, n = 5 x 2 particle groups x 4 time points).

#### Stomach, small intestine, cecum, feces

Due to potential fecal contamination of urine samples in metabolism cages, urine and feces were combined after collected and dried at 60°C overnight. Stomach, small intestine, cecum and dried feces were weighed (±0.1 mg) and microwave digested using Xpress vessels in 10 mL of trace metal grade nitric acid. Microwave digestion (Mars Xpress by CEM Corporation of Matthews, NC, USA) was done using a max temperature control of 200°C with 1600 W of available power. Samples were ramped to 200°C over a period of fifteen minutes and then held at 200°C for fifteen minutes. This microwave cycle was run between 1 and 6 times until samples were completely digested. Digested samples were then placed in 18 mm x 150 mm borosilicate tubes and dried down at 140°C. Hydrogen peroxide (300 μl) was then added to each sample at 125°C and dried down to a final volume of 200 μl at 140°C. Gold was then digested by adding 300 μl of aqua regia, vortexed for 15 seconds, diluted to 6 mL with MilliQ™ water and filtered using 0.22 μm hydrophilic PTFE membrane filters; feces samples were diluted to 50 mL with MilliQ™ water to ensure gold values fell within the standard curve.

#### Brain, heart, lung, spleen, kidney, liver, large intestine, testes, blood

These tissues were weighed (±0.1 mg) and digested with 2 mL of nitric acid in open 16 mm x 150 mm borosilicate tubes at 140°C. Samples were heated for varying lengths of time depending on tissue type until digested. Hydrogen peroxide (300 μl) was then added to each sample at 125°C and dried down to a final volume of 200 μl at 140°C. Gold was then digested by adding 300 μl of aqua regia, vortexed for 15 seconds, diluted to 6 mL with MilliQ™ water and filtered using 0.22 μm hydrophilic PTFE membrane filters.

### Statistical analysis

Sigma Plot 11.0 (Systat Software Inc., Chicago, IL, USA) was used for statistical analysis. All results are presented as averages (n = 5) of the total amount of gold (ng) detected in a given organ, ± the standard deviation. Normality was tested using the Shapiro-Wilk test. All data passed a test of normality and differences between groups were detected using parametric tests. The students *t*-test was used to detect significant differences between two groups (Figure 4), while one-way ANOVA was used to detect differences between several groups (Figure 3). Post-hoc analysis was done using the Bonferroni correction. A p-value < 0.05 was considered statistically significant.

## Abbreviations

PP: Peyer’s patch
GIT: Gastrointestinal tract
TEM: Transmission electron microscopy
ICPMS: Inductively coupled plasma mass spectrometry
PEG: Polyethylene glycol
PTFE: Polytetrafluoroethylene
ddH_2_O: Double distilled water
DMAE: 2-dimethylaminoethanol
LOD: Limit of detection
LOQ: Limit of quantitation
SGF: Simulated gastric fluid
SEM: Scanning electron microscopy
